# Treatment of osteopetrosis combined with fracture around femoral internal fixation: a case report

**DOI:** 10.3389/fsurg.2025.1662541

**Published:** 2025-10-14

**Authors:** Xiaoping Xu, Tao Li

**Affiliations:** 1 Department of Orthopaedics, Shanghai Jiading District Central Hospital, Shanghai, China; 2 Department of Orthopaedics, Xinhua Hospital Affiliated to Shanghai Jiaotong University School of Medicine, Shanghai, China

**Keywords:** osteopetrosis, fracture around internal fixation, intramedullary nail, internal fixation device removal, re-fracture

## Abstract

Osteopetrosis is a rare disease caused by bone resorption disorders due to a decrease in osteoclasts or functional abnormalities, which can easily lead to secondary fractures. Currently, there are few reports on the treatment of fractures around internal fixation in such patients. Here, we report on the treatment experience of a rare case of a patient with osteopetrosis who suffered a fracture around the internal fixation of the right femur. A 59-year-old female patient suffered a right femoral intertrochanteric fracture in 2014 and was diagnosed with osteopetrosis. She underwent right femoral intramedullary nailing surgery. In October 2021, the patient sustained a fracture around the internal fixation device of the right femur due to a fall. We performed surgery to remove the intramedullary nail device and conducted a second internal fixation surgery using a longer intramedullary nail. During follow-up, the patient's recovery was satisfactory. Imaging studies at one year postoperatively showed good healing of the femoral shaft fracture. After more than three years of follow-up, the patient has returned to normal daily activities. Reviewing this case, we found that detailed preoperative planning, appropriate surgical techniques and internal fixation selection, and meticulous intraoperative procedures are key to treating such patients. Additionally, ensuring the safe removal of internal fixation is of critical importance.

## Introduction

Osteopetrosis(OP), also known as marble bone disease or congenital osteosclerosis, was first described by German radiologist Albers-Schonberg in 1904, hence its other name, Albers-Schonberg disease (1). OP is a rare hereditary bone metabolic disease characterised mainly by osteoclast reduction or dysfunction. Excessively high bone density causes brittle bones, which are prone to secondary fractures (2). The exact prevalence of this disease remains unclear, as it is estimated that 40% of patients remain asymptomatic (3). Additionally, the clinical manifestations of this disease lack specificity, making imaging findings a key diagnostic criterion. Currently, there is limited information in the literature regarding treatment methods for fractures in OP patients, with most available data presented in case reports describing the management of postoperative infections, non-union, or fractures around implants (4–6). There are few reports on how to safely remove internal fixation and perform secondary fracture surgery in OP patients after a fracture around the internal fixation. Here, we describe a case of a patient with osteopetrosis who suffered a fracture around the internal fixation of the femur after accidentally falling while walking. x-ray examination showed a fracture around the internal fixation of the right femur, osteopetrosis, and hip arthritis. Further questioning of the patient's medical history revealed that he had suffered a right femoral intertrochanteric fracture in a car accident seven years ago and was treated at a local hospital, where he was diagnosed with OP and severe hip arthritis. However, the patient refused the joint replacement surgery recommended by the doctor at the time and only accepted intramedullary nail internal fixation treatment for the fracture. In this case, a fracture occurred around the intramedullary nail fixation of the femoral shaft. We performed fracture surgery using a long intramedullary nail. This study aims to provide clinical evidence for the development of surgical strategies for similar patients in the future by introducing the experience of safely removing the intramedullary nail and the method of secondary surgery in such patients.

## Case information

**Figure 1:**
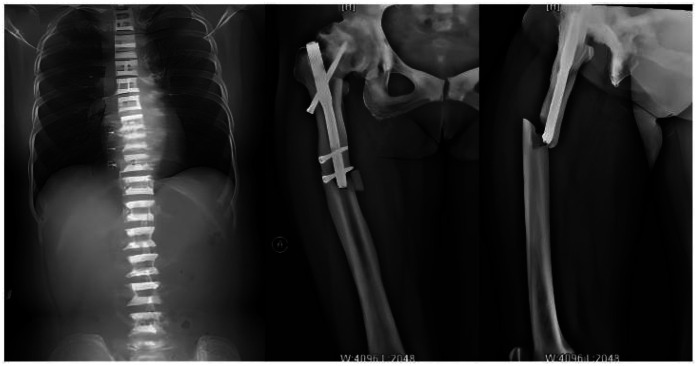
Full-length anteroposterior x-ray film of spine showed that the bone density of upper and lower parts of vertebral body increased, while the density of middle part was lower; preoperative anteroposterior and lateral x-ray images of the right femur show fractures around the internal fixation of the right femur, with signs of hip osteoarthritis and abnormally high bone density.

A 59-year-old woman presented to our emergency department on 5 October 2021 with right thigh pain and limited mobility following an accidental fall while walking. On admission, physical examination revealed significant swelling of the right thigh, visible subcutaneous bruising, marked tenderness on palpation, positive percussion pain, negative pelvic separation compression test, normal range of motion at the knee and ankle joints, and normal peripheral circulation and sensation in the affected limb. To confirm the diagnosis, the patient underwent right femur anteroposterior and lateral x-ray examinations (Figure 1), which showed: Continuity of the cortical bone was interrupted around the internal fixation of the right femoral shaft, and the bone density was abnormally increased, with rough and blurred trabeculae, thickened cortical bone, and narrowed medullary cavity; Full-length anteroposterior x-ray examination of the spine (Figure 1) shows that the density of the upper and lower edges of the vertebral body is increased, and the middle density is lower, showing a “sandwich” change, which is a typical imaging manifestation of osteopetrosis. The clinical diagnosis was: (1) fracture around the internal fixation of the right femur; (2) osteopetrosis; (3) right hip arthritis. Upon further inquiry into the patient's medical history, he reported that he had suffered a right femoral intertrochanteric fracture due to a car accident in 2014, and was also found to have osteopetrosis and hip joint necrosis. At that time, the doctor recommended joint replacement surgery to treat both the fracture and joint necrosis, but the patient refused the surgery because he was young at the time. He then underwent intramedullary nail internal fixation to treat the fracture, which healed well, and he resumed his normal life. The patient has no history of severe internal medical conditions. The patient was referred to our orthopaedic ward for further treatment and is scheduled to undergo fracture surgery.

**Figure 2:**
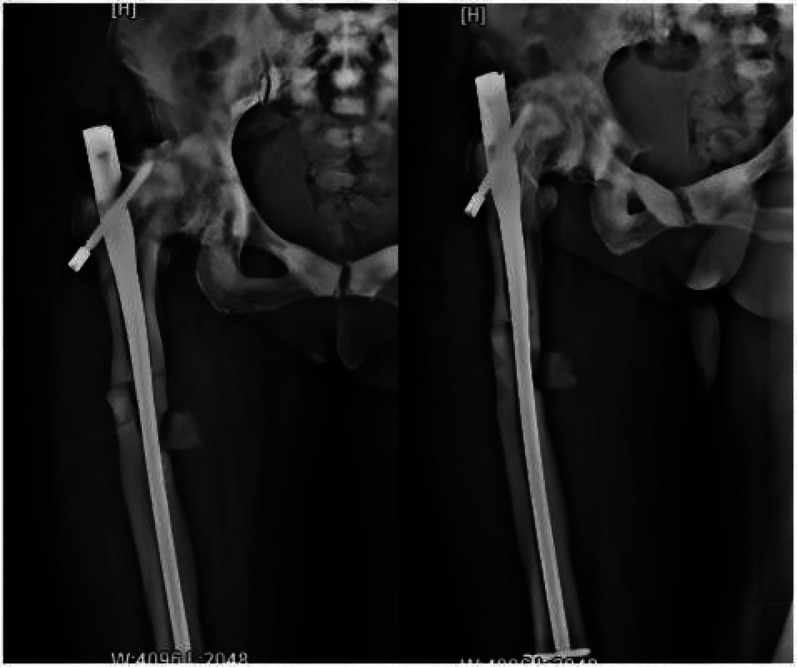
Postoperative x-ray images taken on the first day show that the intramedullary nail device is securely in place, and bone fragments are visible at the fracture site.

**Figure 3:**
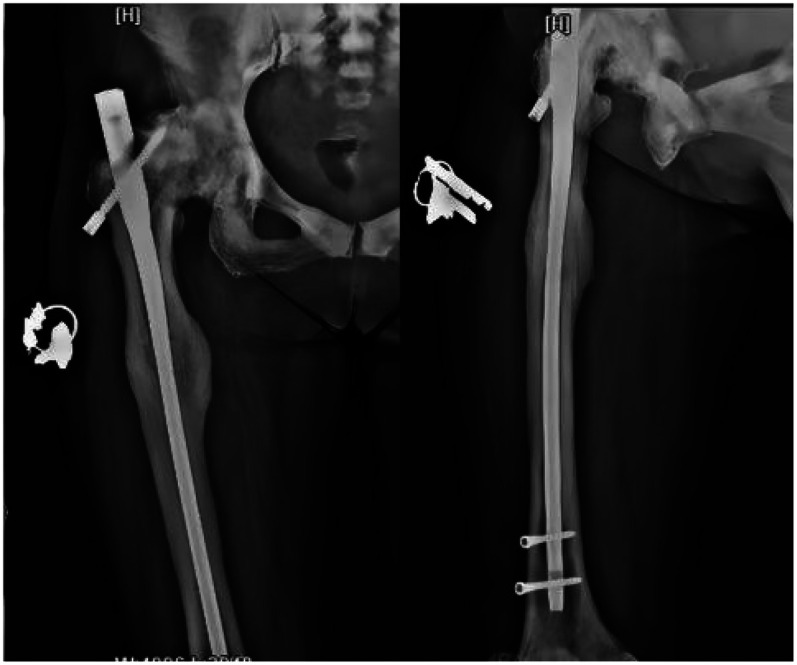
Follow-up x-rays taken one year after surgery showed good fracture healing and no abnormalities in the internal fixation device.

Following admission, the patient received symptomatic treatment including anti-inflammatory and analgesic therapy. Relevant laboratory tests and routine preoperative examinations revealed no significant contraindications for surgery. It was planned to perform a procedure to remove the internal fixation device from the right femoral trochanteric fracture and a closed reduction with intramedullary nail internal fixation for the right femoral fracture. The patient was placed in a supine position on a traction bed. An incision was made along the original surgical scar at the right hip, and the internal fixation device was removed. The apex of the right greater trochanter was then exposed. The needle insertion point was located at the anterior-medial one-third of the greater trochanter apex, slightly medial. The position of the guide wire was verified under fluoroscopy. The fracture was reduced on the traction bed, and the proximal end was dilated to 13 mm. and the distal end was dilated to 11 mm. After confirming the measurements, a 10 mm × 360 mm femoral intramedullary nail was placed. Under fluoroscopy, the fracture reduction was satisfactory. Two distal screws were locked in place using a targeting device, and the fracture ends were tapped and compressed. One proximal locking screw was inserted, and a 0 mm tail cap was installed. The surgery lasted 3.0 h, with an intraoperative blood loss of approximately 260 mL. On the second day postoperatively, a cephalometric and lateral x-ray examination of the femur was performed, with no abnormalities detected (Figure 2). The patient was discharged five days later, and was instructed to take anti-osteoporosis treatment after discharge, and isometric contraction of quadriceps femoris and ankle pump exercise were performed in bed 1–2 weeks after operation. 2–6 weeks after operation, straight leg lifting training, hip joint mobility training and partial weight-bearing exercise with walker were carried out; Six weeks after the operation, the patient changed to walking with one leg to carry out daily life training. One year after the operation, the x-ray examination showed that the fracture of femoral cadres healed well (Figure 3). During a follow-up period of over three years, the patient exhibited no significant neurological or motor dysfunction and no other complications were observed. The quality of life of patients has been greatly improved.

## Discussion

OP is a heterogeneous group of rare genetic bone diseases characterized by reduced osteoclast activity, increased bone mass, and high bone fragility (7). Most patients are typically hospitalised due to fractures, with the most common site being the femur (8). Currently, most patients with OP who suffer from fractures are recommended to undergo internal fixation surgery, which can provide firm fixation for the fracture and enable patients to start functional rehabilitation training at an early stage, which is very beneficial for fracture healing and functional recovery (9). Although postoperative complications still plague us, with advances in surgical techniques and the accumulation of clinical experience in the treatment of OP, the incidence of complications has also been significantly reduced (10). According to the research of Ding H's research group (11) and Lamut A's research group (12), in the case reports of OP published before 2005, the incidence of surgery-related complications was 54.55%, the reoperation rate was 27.27%, and the non-healing rate was 18.18%. In contrast, in cases reported after 2005, the complication rate decreased to 21.05%, the reoperation rate decreased to 10.53%, and the non-union rate decreased to 0%. These findings demonstrate that as more reports on such challenging cases emerge, the development of related diagnostic and therapeutic approaches, as well as safe and effective techniques, will accelerate. This article introduces the diagnosis and treatment experience of a patient with OP who suffered a fracture around the internal fixation of the right femur, and hopes to provide some clinical reference for the future treatment of such patients.

Fracture surgery in patients with OP is a major challenge for orthopaedic surgeons due to the abnormal bone strength and fragility, especially when the surgery involves extensive medullary expansion and drilling, which can easily lead to drill bit fracture or intraoperative fracture. Therefore, the choice of surgical method is crucial (13, 14). In this case, the patient had previously undergone intramedullary nail surgery, and the medullary cavity of the femur had not completely disappeared, allowing for medullary reaming. Therefore, we opted for long intramedullary nail treatment. Previous literature has reported on the technical challenges of using intramedullary nails to treat fractures in such patients, emphasising that opening the intramedullary canal is difficult and that care must be taken to avoid drill bit damage or intraoperative fractures (15, 16). Therefore, new drill bits should be used for each marrow expansion or drilling operation. The action should be steady and moderate, not violent. Abnormal bone density means that we need to use a continuous supply of physiological saline to cool down the drill bit when drilling holes for the hip screw and the distal locking screw, to prevent friction heat. The drill bit must also be kept stable during drilling to prevent the force of the reaction from changing the direction of the drill bit.We combined the recommendations proposed by the Kent J group (17), the Bhargava A group (18), and the Beckers G group (19), and based on our own experience, we summarised the following operational methods: First, start medullary expansion with a smaller drill bit and gradually increase the drill bit size; Second, maintain a constant speed during medullary expansion and avoid rapid up-and-down movements; Third, continuously cool the drill bit with saline and remove bone debris to prevent friction heat from causing bone burns or drill bit damage/fracture. If possible, use a new sharp drill bit for each drilling session; Fourth, avoid using excessive force or hammering when inserting the main nail; finally, ensure that the screw hole has been fully drilled before inserting the distal locking nail. This method does not impose stringent requirements on drill bit material and is simple to perform, making it highly versatile. Of course, if conditions permit, we suggest using cemented carbide bits made of superhard materials such as tungsten carbide. It would be great if diamond coated bits could be used, which would greatly save the operation time.

Another challenge in this case lies in safely removing the intramedullary nail system without causing screw fracture or secondary damage to the fracture ends. We reviewed the relevant literature and found no clear reports detailing specific surgical techniques. Based on our experience, we established the sequence for removing the internal fixation prior to surgery and emphasised maintaining stability of the fracture ends during the procedure. During surgery, we first removed the tail cap, inserted the extractor into the intramedullary nail, and used it to stabilise the main nail; then we removed the distal locking screw, loosened the other screw; next, we removed the proximal locking screw, followed by the final distal screw; finally, we tapped the intramedullary nail outward, applying moderate force, and once it felt loose, we tapped it out at a steady pace. We know that the abnormal bone density of patients with OP increases the risk of internal fixation removal. In order to loosen the screws, improper force may cause screw fracture or intraoperative fracture, which greatly increases the difficulty of subsequent internal fixation surgery (20). Therefore, detailed preoperative planning and meticulous intraoperative manipulation are essential. These measures can effectively prevent intraoperative accidents and reduce surgery time.

This case required a long surgery time, mainly due to the difficulty of locating the tail cap and the broken end screw when removing the internal fixation, and the time-consuming nature of the expansion of the medullary canal and drilling. Due to the patient having undergone surgery seven years ago, we found that the tail cap had been covered by bone tissue when we searched for it. Additionally, as the fracture occurred at the distal locking screw, it was displaced from its original position, making it difficult to locate. We used a K-wire to assist with the positioning and, under fluoroscopy, determined their locations before removing them. Furthermore, when expanding the medullary canal or drilling, it is essential to use a new drill bit each time, performing the action at a steady and moderate speed. The abnormal bone density also necessitated the use of a continuous supply of physiological saline to cool down the drill bit during drilling at the hip screw and distal locking screw, to prevent friction heat. Additionally, the drill bit must be kept stable during drilling to prevent the force of the reaction from changing the direction of the drill bit. Similarly, after the surgery, the area must be thoroughly flushed with a large amount of physiological saline to prevent infection. While these steps are time-consuming, they are essential.The operation in this case took a long time. This was mainly due to the difficulty of finding the tail cap and the broken end screw when removing the internal fixation, as well as the time-consuming expansion of the marrow cavity and drilling during the placement of the long intramedullary nail. As this patient had not had surgery for seven years, we found that the tail cap had been covered by bone tissue when we searched for it. Additionally, as the fracture occurred at the distal locking screw, it was displaced from its original position, making it difficult to find. We finally located them using a K-wire to assist with the positioning and confirmed their location with intraoperative fluoroscopy before removing them.

This case has some limitations, namely that the position of the proximal screw is displaced and relatively unstable. This is because the patient's femoral head, neck and trochanter have undergone abnormal morphological changes, resulting in the discovery of the main screw being slightly longer than the marrow cavity after completing the surgical enlargement. At this point, it was no longer possible to change the main screw specifications, which caused the hip screw holes on the main screw to shift towards the proximal end. Despite our efforts to control the direction of the hip screw holes towards the distal end during drilling, the results were not entirely satisfactory. Fortunately, during the operation, we discovered that although the hip screw was displaced towards the proximal end, it had not penetrated the femoral head and the fracture ends were adequately fixed. To avoid further harm to the patient from repeated procedures, we ended the surgery. During subsequent follow-ups, we found that the patient's fracture had healed well, with no significant change in the position of the hip screw and no penetration of the femoral head, and no evidence of damage to the femoral head.

## Conclusion

This report introduces a case of a patient with osteopetrosis who suffered a fracture around the internal fixation device of the femur. It aims to illustrate how to safely remove the internal fixation device and effectively perform secondary fracture surgery. It finds that detailed preoperative planning, reasonable surgical methods and internal fixation choices, as well as meticulous intraoperative operations can effectively reduce surgical time, difficulty and complications. Therefore, when encountering such patients in the future, orthopaedic surgeons should conduct thorough preoperative preparations and develop detailed surgical plans to control treatment risks and achieve better treatment outcomes for patients.

## Data Availability

The original contributions presented in the study are included in the article/Supplementary Material, further inquiries can be directed to the corresponding author.
